# Supplementary material for: Endo-lysosomal assembly variations among human leukocyte antigen class I (HLA class I) allotypes
Source: eLife. 2023 Feb 1;12:e79144. doi: 10.7554/eLife.79144 (PMC9917446; doi:10.7554/eLife.79144)
Supplement: Supplementary file 1. — Donors were selected from our previously described cohort of HLA genotyped healthy participants (Yarzabek et al., 2018). Ten primary groups were recruited for this study. Groups 1–8 were selected to be either homozygous for an HLA-B allotype of interest, or heterozygous for one Bw6 allotype and one Bw4 allotype so that anti-Bw6 and anti-Bw4 monoclonal antibodies could be utilized to measure the specific expression/localization of individual HLA-B allotypes. Donors in these groups were selected to have one or less HLA-C allotype that cross-reacts with the anti-Bw6 antibody, or no HLA-A allotypes that cross-react with the anti-Bw4 antibody (Yarzabek et al., 2018). Two cross-reactive HLA-C allotypes (denoted by asterisks) are permitted if the donor is homozygous for the HLA-B allotype of intertest, or if assays not using anti-Bw6 were not performed (e.g. peptide receptivity assays with the HC10 monoclonal antibody). Group 9 donors expressed B*08:01, B*35:01, or both and were selected for use as effector antigen-specific CTLs for all T cell activation assays. Heterozygosity for Bw6/Bw4 was not required for studies with this group. Group 10 contained donors heterozygous for B*08:01 and B*35:01 and were used as antigen-presenting cells for cross-presentation assays. For assays where specific HLA genotype was not required, such as DQ-Ova experiments, non-genotyped donors 248–275 were used, or blood was obtained from the University of Michigan Platelet Core. Donors from the Platelet Core are labeled. [file elife-79144-supp1.docx]

**Supplementary File 1**

| **GROUP 1** | **B*15:01^+^ donors: B*15:01/Bw4 heterozygous** | | | | | | |
| --- | --- | --- | --- | --- | --- | --- | --- |
|  | **Donor ID** | **HLA-A** | **HLA-A** | **HLA-B (Bw6)** | **HLA-B (Bw4)** | **HLA-C** | **HLA-C** |
|  | 124 | A*32:01:01 | A*11:01:01:01 | B*15:01:01:01 | B*53:01:01 | C*06:02:01:01 | C*04:01:01:01 |
|  | 128 | A*11:01:01:01 | A*02:01:01:01 | B*15:01:01:01 | B*44:02:01:01 | C*03:03:01:01 | C*05:01:01:02 |
|  | | | | | | | |
| **GROUP 2** | **B*07:02^+^ donors: either B*07:02 double positive, or B*07:02/Bw4 heterozygous** | | | | | | |
|  | **Donor ID** | **HLA-A** | **HLA-A** | **HLA-B (Bw6)** | **HLA-B (Bw4)** | **HLA-C** | **HLA-C** |
|  | 14 | A*03:01:01:01 | A*02:01:01:01 | B*07:02:01 | B*51:01:01:01 | C*15:02:01:01 | C*07:02:01:03 |
|  | 20 | A*02:01:01:01 | A*02:01:01:01 | B*07:02:01 | B*37:01:01 | C*07:02:01:03 | C*06:02:01:01 |
|  | 196 | A*02:01:01:01 | A*03:01:01:01 | B*07:02:01 | B*44:02:01:01 | C*05:01:01:02 | C*07:02:01:03 |
|  | 250 | A*26:01:01:01 | A*31:01:02:01 | B*07:02:01 | B*27:05:02:09 | C*07:02:01:03 | C*02:02:02:01 |
|  | 269 | A*03:01:01:01 | A*03:01:01:01 | B*07:02:01 | B*57:01:01:01 | C*06:02:01:01 | C*07:02:01:03 |
|  | 270 | A*02:01:01:01 | A*02:01:01:01 | B*07:02:01 | B*07:02:01 | C*07:02:01:03 | C*07:02:01:03 |
|  | | | | | | | |
| **GROUP 3** | **B*44:02^+^ donors: B*44:02/Bw6 heterozygous** | | | | | | |
|  | **Donor ID** | **HLA-A** | **HLA-A** | **HLA-B (Bw4)** | **HLA-B (Bw6)** | **HLA-C** | **HLA-C** |
|  | 94 | A*68:01:02:01 | A*01:01:01:01 | B*44:02:01:01 | B*08:01:01 | C*07:01:01:01 | C*05:01:01:01 |
|  | 128 | A*11:01:01:01 | A*02:01:01:01 | B*44:02:01:01 | B*15:01:01:01 | C*03:03:01:01 | C*05:01:01:02 |
|  | 196 | A*02:01:01:01 | A*03:01:01:01 | B*44:02:01:01 | B*07:02:01 | C*05:01:01:02 | C*07:02:01:03 |
|  | 267 | A*68:01:02:01 | A*02:01:01:01 | B*44:02:01:01 | B*07:02:01 | C*07:04:01:01 | C*07:02:01:03 |
|  | | | | | | | |
| **GROUP 4** | **B*51:01^+^ donors: B*51:01/Bw6 heterozygous** | | | | | | |
|  | **Donor ID** | **HLA-A** | **HLA-A** | **HLA-B (Bw4)** | **HLA-B (Bw6)** | **HLA-C** | **HLA-C** |
|  | 14 | A*03:01:01:01 | A*02:01:01:01 | B*51:01:01:01 | B*07:02:01 | C*15:02:01:01 | C*07:02:01:03 |
|  | 232 | A*02:01:01:01 | A*02:01:01:01 | B*51:01:01:01 | B*07:02:01 | C*14:02:01:01 | C*07:02:01:03 |
|  | | | | | | | |
| **GROUP 5** | **B*57:01^+^ donors: B*57:01/Bw6 heterozygous** | | | | | | |
|  | **Donor ID** | **HLA-A** | **HLA-A** | **HLA-B (Bw4)** | **HLA-B (Bw6)** | **HLA-C** | **HLA-C** |
|  | 156 | A*01:01:01:01 | A*03:01:01:01 | B*57:01:01 | B*14:02:01:01 | C*07:01:01:01 | C*05:01:01:01 |
|  | 178 | A*01:01:01:01 | A*02:01:01:01 | B*57:01:01 | B*08:01:01 | C*06:02:01:01 | C*07:01:01:01 |
|  | 210 | A*01:01:01:01 | A*03:01:01:01 | B*57:01:01 | B*35:01:01:02 | C*06:02:01:01 | C*04:02:01:01 |
|  | 269 | A*03:01:01:01 | A*03:01:01:01 | B*57:01:01 | B*07:02:01 | C*06:02:01:01 | C*07:02:01:03 |
|  | | | | | | | |
| **GROUP 6** | **B*27:05^+^ donors: B*27:05/Bw6 heterozygous** | | | | | | |
|  | **Donor ID** | **HLA-A** | **HLA-A** | **HLA-B (Bw4)** | **HLA-B (Bw6)** | **HLA-C** | **HLA-C** |
|  | 142 | A*02:01:01:01 | A*26:01:01:01 | B*27:05:02 | B*40:01:02 | C*03:04:01:01 | C*01:02:01:01 |
|  | 250 | A*26:01:01:01 | A*31:01:02:01 | B*27:05:02:09 | B*07:02:01 | C*07:02:01:03 | C*02:02:02:01 |
|  | 256 | A*02:01:01:01 | A*02:01:01:01 | B*27:05:02:05 | B*07:02:01 | C*07:02:01:03 | C*03:03:01:01 |
|  | | | | | | | |
| **GROUP 7** | **B*08:01^+^ donors: either B*08:01 double positive, or B*08:01/Bw4 heterozygous** | | | | | | |
|  | **Donor ID** | **HLA-A** | **HLA-A** | **HLA-B (Bw6)** | **HLA-B (Bw4 or B*08:01)** | **HLA-C** | **HLA-C** |
|  | 9 | A*01:01:01:01 | A*01:01:01:01 | B*08:01:01 | B*08:01:01 | C*07:01:01:01 | C*07:01:01:01 |
|  | 28 | A*01:01:01:01 | A*02:01:01:01 | B*08:01:01 | B*51:01:01:01 | C*07:01:01:01 | C*15:13 |
|  | 55 | A*01:01:01:01 | A*23:01:01 | B*08:01:01 | B*44:03:01 | C*07:01:01:01 | C*04:09 |
|  | 94 | A*01:01:01:01 | A*68:01:02:01 | B*08:01:01 | B*44:02:01:01 | C*07:01:01:01 | C*05:01:01:02 |
|  | 105* | A*01:01:01:01 | A*26:01:01:01 | B*08:01:01 | B*38:01:01 | C*07:01:01:01 | C*12:03:01:01 |
|  | 121 | A*01:01:01:01 | A*01:01:01:01 | B*08:01:01 | B*27:05:02 | C*07:01:01:01 | C*02:07 |
|  | 130 | A*01:01:01:01 | A*30:01:01 | B*08:01:01 | B*13:02:01 | C*07:01:01:01 | C*06:02:01:01 |
|  | 137 | A*01:01:01:01 | A*01:01:01:01 | B*08:01:01 | B*37:01:01 | C*07:01:01:01 | C*06:02:01:01 |
|  | 148* | A*01:01:01:01 | A*02:01:01:01 | B*08:01:01 | B*49:01:01 | C*07:01:01:01 | C*07:01:01:01 |
|  | 166 | A*01:01:01:01 | A*01:01:01:01 | B*08:01:01 | B*08:01:01 | C*07:01:01:01 | C*07:01:01:01 |
|  | 178 | A*01:01:01:01 | A*02:01:01:01 | B*08:01:01 | B*57:01:01 | C*07:01:01:01 | C*06:02:01:01 |
|  | 237 | A*68:01:01:02 | A*02:01:01:01 | B*08:01:01 | B*44:02:01:01 | C*07:01:01:01 | C*05:01:01:02 |
|  | | | | | | | |
| **GROUP 8** | **B*35:01^+^ donors: either B*35:01 double positive, or B*35:01/Bw4 heterozygous** | | | | | | |
|  | **Donor ID** | **HLA-A** | **HLA-A** | **HLA-B (Bw6)** | **HLA-B (Bw4)** | **HLA-C** | **HLA-C** |
|  | 24 | A*02:01:01:01 | A*24:02:01:01 | B*35:01:01:02 | B*51:01:01:01 | C*15:02:01:01 | C*04:04:01 |
|  | 136 | A*11:01:01:01 | A*30:01:01 | B*35:01:01:02 | B*13:02:01 | C*04:01:01:01 | C*06:02:01:01 |
|  | 141 | A*02:01:01:01 | A*03:01:01:01 | B*35:01:01:02 | B*44:02:01:01 | C*05:01:01:02 | C*04:01:01:01 |
|  | 168 | A*02:01:01:01 | A*11:01:01:01 | B*35:01:01:02 | B*51:01:01:01 | C*15:02:01:01 | C*04:01:01:01 |
|  | 187 | A*01:01:01:01 | A*02:01:01:01 | B*35:01:01:02 | B*44:02:01:01 | C*05:01:01:02 | C*04:01:01:05 |
|  | 210 | A*01:01:01:01 | A*03:01:01:01 | B*35:01:01:02 | B*57:01:01 | C*06:02:01:01 | C*04:01:01:01 |
|  | 274 | A*33:03:01:01 | A*32:01:01:01 | B*35:01:01:05 | B*44:03:01:01 | C*14:03:01:01 | C*04:01:01:14 |
|  | | | | | | | |
| **GROUP 9** | **B*08:01^+^ or B*35:01^+^ donors for antigen-specific CTL expansion** | | | | | | |
|  | **Donor ID** | **HLA-A** | **HLA-A** | **HLA-B** | **HLA-B** | **HLA-C** | **HLA-C** |
|  | 9 | A*01:01:01:01 | A*01:01:01:01 | B*08:01:01 | B*08:01:01 | C*07:01:01:01 | C*07:01:01:01 |
|  | 16 | A*01:01:01:01 | A*02:01:01:01 | B*08:01:01 | B*35:01:01:02 | C*07:01:01:01 | C*04:01:01:06 |
|  | 40 | A*03:01:01:01 | A*11:01:01:01 | B*35:01:01:02 | B*35:03:01 | C*04:01:01:01 | C*04:01:01:01 |
|  | | | | | | | |
| **GROUP 10** | **B*08:01/B*35:01 double positive donors for cross-presentation APCs** | | | | | | |
|  | **Donor ID** | **HLA-A** | **HLA-A** | **HLA-B** | **HLA-B** | **HLA-C** | **HLA-C** |
|  | 16 | A*01:01:01:01 | A*02:01:01:01 | B*08:01:01 | B*35:01:01:02 | C*07:01:01:01 | C*04:01:01:06 |
|  | 25 | A*01:01:01:01 | A*02:01:01:01 | B*08:01:01 | B*35:01:01:02 | C*07:01:01:01 | C*04:01:01:06 |
|  | 132 | A*01:01:01:01 | A*24:02:01:01 | B*08:01:01 | B*35:01:01:02 | C*07:01:01:01 | C*07:01:01:01 |

**Supplementary Table 1. Healthy human donors and HLA genotypes used in study.**

Donors were selected from our previously described cohort of HLA genotyped healthy participants (Yarzabek et al., 2018). Ten primary groups were recruited for this study. Groups 1-8 were selected to be either homozygous for an HLA-B allotype of interest, or heterozygous for one Bw6 allotype and one Bw4 allotype so that anti-Bw6 and anti-Bw4 monoclonal antibodies could be utilized to measure the specific expression/localization of individual HLA-B allotypes. Donors in these groups were selected to have one or less HLA-C allotype that cross-reacts with the anti-Bw6 antibody, or no HLA-A allotypes that cross-react with the anti-Bw4 antibody (Yarzabek et al., 2018). Two cross-reactive HLA-C allotypes (denoted by asterisks) are permitted if the donor is homozygous for the HLA-B allotype of intertest, or if assays not using anti-Bw6 were not performed (for example, peptide receptivity assays with the HC10 monoclonal antibody). Group 9 donors expressed B*08:01, B*35:01, or both, and were selected for use as effector antigen-specific CTLs for all T cell activation assays. Heterozygosity for Bw6/Bw4 was not required for studies with this group. Group 10 contained donors heterozygous for B*08:01 and B*35:01 and were used as antigen-presenting cells for cross-presentation assays. For assays where specific HLA genotype was not required, such as DQ-Ova experiments, non-genotyped donors 248-275 were used, or blood was obtained from the University of Michigan Platelet Core. Donors from the Platelet Core are labeled with the prefix “PCD”.

YARZABEK, B., ZAITOUNA, A. J., OLSON, E., SILVA, G. N., GENG, J., GERETZ, A., THOMAS, R., KRISHNAKUMAR, S., RAMON, D. S. & RAGHAVAN, M. 2018. Variations in HLA-B cell surface expression, half-life and extracellular antigen receptivity. *Elife,* 7.
